# Relationship between aging and excess body fat with markers of inflammation, skeletal muscle mass and strength in Mexican community-dwelling people

**DOI:** 10.1007/s11845-024-03727-0

**Published:** 2024-06-04

**Authors:** Víctor Manuel Mendoza-Núñez, Jimena Valeria Aguilar-Curiel, Lilia Castillo-Martínez, Wendy Daniella Rodríguez-García, Nayeli Vaquero-Barbosa, Juana Rosado-Pérez, Taide Laurita Arista-Ugalde

**Affiliations:** 1 Research Unit On Gerontology, FES Zaragoza, National Autonomous University of Mexico, Guelatao # 66, Alcaldía Iztapalapa, 09230 Mexico City, Mexico; 2 Clinical Nutrition Department, Instituto Nacional de Ciencias Médicas y Nutrición Salvador Zubirán, Mexico City, Mexico; 3 Nutrition Career, FES Zaragoza, National Autonomous University of Mexico, 09230 Mexico City, Mexico

**Keywords:** Aging, Chronic inflammation, Muscle mass, Obesity, Sarcopenia, Strength

## Abstract

**Introduction:**

Aging is accompanied by changes in body composition, such as an increase in fat mass (FM), a decrease in skeletal muscle mass index (SMMI) and muscle strength, combined with a chronic inflammatory process (CI).

**Objective:**

Determine the relationship between age and excess body fat with markers of chronic inflammation, skeletal muscle mass and strength.

**Methods:**

A cross-sectional alitical study was carried out in a convenience sample of adults 45 to 59 years old (*n* = 100) and older adults 60 to 74 years old (*n* = 133). All participants had their body composition measured with an impedance meter. They were subsequently divided into two groups: (i) with excess fat (WEF), (ii) without excess fat (NEF), in order to relate excess fat and age with inflammation, muscle mass and strength.

**Results:**

NEF adults and older adults had similar values of SMMI (9.1 ± 1.5 vs. 8.8 ± 1.3, *p* > 0.05) and strength (28 ± 8 vs. 27 ± 8.6, *p* > 0.05). Likewise, WEF adults showed significantly lower values than NEF adults in the SMMI (7.9 ± 0.8 vs. 9.1 ± 1.5, *p* < 0.05) and strength (28 ± 8 vs. 22 ± 5, *p* < 0.001). Also, WEF older adults presented significantly lower values in the SMMI (15.9 ± 1.8 vs. 22.8 ± 5.1, *p* < 0.05) and strength (17.9 ± 4.8 vs. 27 ± 8.6, *p* < 0.001).

**Conclusions:**

Our findings suggest that excess fat mass is a risk factor that has a significantly greater influence than aging per se on the index of skeletal muscle mass and strength.

## Introduction

Although there is no universally agreed upon concept of human aging [1–3]. Our research group defines it as a “gradual and adaptive process, characterized by a relative decrease in the reserve and biological response to the demands to maintain or recover homeostasis, due to molecular, biochemical, physiological, morphological, psychological and social modifications, caused by the genetic load and the accumulated wear and tear faced by the challenges that the person faces throughout their history in a certain environment, which is manifested by individualized physical, psychological and social changes” [4, 5].

One of the main diseases related to aging is sarcopenia, which is defined as “progressive and generalized skeletal muscle disorder that is associated with increased likelihood of adverse outcomes including falls, fractures, physical disability and mortality” [6]. Sarcopenia is considered a muscle disease, in which low muscle strength outweighs the role of low muscle mass as the main determinant of its repercussions. This disease has been associated with old age, without considering that its development begins from earlier stages, especially there are risk factors such as inadequate diet, sedentary lifestyle and obesity [6].

In this framework, for the purposes of clinical and community intervention we assume that the beginning of human aging is in the fifth decade of life (around 45 years), as noted by Lemoine (2020) [1]. However, we recognize that physical changes in organs and systems are asynchronous, as well as individualized. In this regard, among the most important changes during aging is a decrease in skeletal muscle mass (SMM) between 3 to 8% per decade starting at age 30 [7]. Likewise, after the age of 60, there is a 0.5% decrease in annual weight, a loss of 1 cm in height for each decade of life and an increase in fat mass, which favors the appearance of sarcopenic obesity linked to a chronic inflammatory process [8, 9].

Obesity causes a permeability of fat mass (FM) to skeletal muscle due to the production of fatty acids that are deposited in some tissues including skeletal muscle, thus reducing its quality and function. This promotes lipotoxicity, which is an effect characterized by a dysfunction of the functional properties and contractility of muscle fibers, thus causing a decrease in muscle strength and power [10–12]. For this reason, the purpose of the present study is to determine the relationship between age and excess body fat mass with the markers of CI, SMM and strength in people between 45 and 74 years of age.

## Methods

### Study design and participants

After informed consent, a cross-sectional analytical study was carried out in a convenience sample of 233 people ≥ 45 years of age from Mexico City: (i) adults 45–59 years old n = 100; (ii) older adults 60 to 74 years old n = 133. The project was registered in ISRCTN-48485253 and approved by the Ethics Committee of the Faculty of Higher Studies Zaragoza, UNAM (FESZ/DEPI/CI/039/20).

The study variables were: obesity, fat mass, skeletal muscle mass, strength and inflammation markers (IL1, IL6, IL8, IL10 and TNF-α).

Tthe groups of adults and older adults were subdivided as: (i) with excess fat (WEF) and (ii) not excess fat (NEF), considering the values of the cut-off points of the first and second tertile of FM (%). With the aim of being able to relate excess fat with inflammation, muscle mass and strength.

### Anthropometric measurements

To determine the body dimensions of the patients, weight measurements and waist circumference were performed. For body weight, a calibrated medical scale (SECA, Hamburg, Germany) and for carving a wall stadiometer (SECA, Hamburg, Germany) were used; for this purpose, patients were asked to place their heels together, their heads upright in the Frankfort plane and contact with the stadiometer. The distribution of abdominal fat was obtained by measuring waist circumference with the help of a medical tape measure (SECA, Hamburg, Germany), which was placed at the navel level. All measurements were made by trained personnel from FES-Z [13].

### Body composition

Body composition was measured by the single-frequency bioelectric impedance method with the RJL® equipment. For this measurement, patients had to be fasting and not have metal objects, to avoid interference with the electrical frequencies generated by the impedance meter. They were asked to lie supine on a flat surface and then four electrodes were placed on them, two on the hand and two on the foot on the right side. Subsequently, resistance (R) and reactance (Xc) data were obtained considering height, weight and age. Fat mass (FM) and skeletal muscle mass (SMM) were measured [14].

### Muscle strength

To measure muscle strength, a Jamar hydraulic dynamometer adjustable to the width of the hand was used with a measurement range of 0 to 100 kg. The participant had to stand with his arms extended parallel to the trunk, the dynamometer was held in his hand and without support he was asked to exert maximum force. 3 alternating measurements were obtained from each arm, allowing 1 min to rest between each one, the value of each measurement was noted and the maximum value was taken into account.

### Blood Sampling and biochemical analyses

Glucose, urate, albumin, cholesterol, triglycerides, and HDL-C concentration levels were determined using a Merck Vitalab Eclipse autoanalyzer® (Merck, Dieren, The Netherlands). In particular, glucose levels were measured by the glucose oxidase method, and urate levels by the uricase colorimetric method. Albumin levels were measured with the bromocresol green technique. The low-density lipoproteins (LDL cholesterol) were calculated by the Friedewald equation: LDL = total cholesterol − (triglycerides/5 + HDL) [15].

### Inflammatory cytokines

Cytokine concentrations were measured using the BD Human Inflammatory Cytokines Cytometric Bead Array (CBA) kit and technique (BD Biosciences, San Jose, CA, USA). Quantifications were performed using the flow cytometric method for measurement and the FCAP ArrayTM software v3.0 for conversion to pg/mL. Aliquots of serum samples were assayed by flow cytometry (CBA Kit, Human Inflammatory Cytokine, BD, San Diego, CA, USA) to determine the levels of IL: IL1-β, IL-6, IL-8, IL-10, and tumor necrosis factor-alpha (TNF-α). For the measurement of CRP, particles coated with anti-human CRP antibodies were used, which were agglutinated by CRP molecules present in the serum samples analyzed. Since the agglutination causes changes in the absorbance proportionally to the concentration of CRP and after comparison with a calibrator, it was possible to determine the exact concentration of the protein. This test was carried out on the Selectra Junior automated equipment (Vital Scientific, Dieren, the Netherlands) under a turbidimetric principle, using a commercial kit from Spinreact (CRP Turbi 1107101L; Girona, Spain) [16].

### Data analysis

The data obtained were reported as mean and standard deviation and percentages. The population was divided into tertiles in terms of the percentage of fat mass in order to categorize them as: (i) Not Excess Fat (NEF) Tertile 1, values less than or equal to the cut-off point of the 33 percentile of FM (%); (ii) With Excess Fat (WEF) Tertile 3, values greater than or equal to the 66 percentile cut-off point of FM (%). Student's t was used for normal data and Mann–Whitney U for non-parametric data. *p* < 0.05 is considered significant. For the comparison between groups according to age and tertiles of percentage of fat mass, a one-way ANOVA was performed to compare means. The SPSS V20 statistical package (Armonk, NY, USA) was used.

## Results

### Clinical, anthropometric and biochemical characteristics

The biochemical parameters by study group showed that older adults had significantly higher blood concentrations of total cholesterol, HDL and LDL than the adult group (p < 0.001) (Table 1). Likewise, adults group had greater weight, height, strength and skeletal muscle mass index (SMMI) than older adults gropu (*p* < 0.05), in contrast the group of older adults had higher fat mass (%) than the group of adults (*p* < 0.001) (Table 2).

**Table 1:** Biochemical parameters by age group

Variables	Adults *n* = 100	Older adults *n* = 133	*p value*
Glucose (mg/dL)	92 (84 – 106)	96 (88 – 116)	0.078
Urea (mg/dL)	27 (23 – 32.3)	31 (27 – 36.8)	< 0.001
Creatinine (mg/dL)	0.8 (0.7 – 0.87)	0.8 (0.8 – 0.9)	0.862
Uric acid (mg/dL)	4.65 (3.60 – 5.88)	3.95 (3.5 – 5.05)	0.055
Cholesterol (mg/dL)	187 ± 42	204 ± 44	< 0.001
LDL (mg/dL)	105 ± 35	121 ± 42	< 0.01
HDL (mg/dL)	46 (38 – 53)	53 (44 – 63)	< 0.001
Triglycerides (mg/dL)	139 (112 – 182)	150 (109 – 197)	0.527
Hemoglobin (mg/dL)	15.1 (14 – 16.4)	15 (13.9 – 16.3)	0.556
Hematocrit (%)	44.5 (41 – 47.3)	45 (42 – 47)	0.651
Albumin (mg/dL)	4.4 (4.1 – 4.5)	4.4 (4.2 – 4.6)	0.935

*HDL *High density lipoprotein, *LDL* Low density lipoprotein

Data are presented as mean ± SD and median (p25 – p75). Adults correspond to the group from 45 to 59 years old and Older adults to the group from 60 to 74 years old. For comparison, Student's t was used for normal data and Mann–Whitney U for non-parametric data. *p* < 0.05 is considered significant

**Table 2:** Clinical and anthropometric characteristics by age group

Variables	Adults *n* = 100	Older dults *n* = 133	*p value*
Weight (kg)	76.6 ± 14.6	72 ± 11.6	< 0.01
Height (cm)	157 ± 7.2	155 ± 8.1	< 0.05
FM (%)	43.8 (39.3 – 47.3)	48.6 (43.8 – 52.7)	< 0.001
FM (kg)	31.7 (26.7 – 37.6)	34.3 (28.6 – 38.3)	0.103
SMMI (kg/m^2^)	8.18 (7.52 – 895)	7.61 (6.75 – 8.59)	< 0.01
BMI (kg/m^2^)	31.1 ± 4.9	30.2 ± 4.7	0.179
WC (cm)	99 (90.5 – 107)	99 (90 – 107)	0.885
Strength (kg)	22 (20 – 28)	20 (18 – 26)	< 0.01

*FM* fat mass, *SMMI* skeletal muscle mass index, *BMI* body mass index, *WC* waist circumference

Data are presented as mean ± SD and median (p25 – p75). Adults correspond to the group from 45 to 59 years old and Older adults to the group from 60 to 74 years old. For comparison, Student's t was used for normal data and Mann–Whitney U for non-parametric data. *p* < 0.05 is considered significant

Regarding inflammation markers, it was observed that the concentration of IL-1 was significantly higher in adults compared to older adults [14.7 (8.7 – 15.6) vs. 9.19 (7.2 –10.8), *p* < 0.001] (Table 3).

**Table 3:** Markers of inflammation by age group

Variables	Adults *n* = 100	Older adults *n* = 133	*p value*
IL-1 ($\mu g/mL$)	14.3 (8.7 – 15.6)	9.19 (7.2 – 10.8)	< 0.001
IL-6 ($\mu g/mL$)	6.46 (5.67 – 7.85)	7.39 (5.02 – 9.42)	0.455
IL-8 ($\mu g/mL$)	16.9 (13.7 – 26.9)	15.8 (12.3 – 40.4)	0.742
IL-10 ($\mu g/mL$)	4.04 (3.47 – 4.32)	3.90 (1.57 – 4.91)	0.582
TNF-α ($\mu g/mL$)	6.08 (4.87 – 6.48)	6.29 (2.78 – 8.53)	0.195

*Abbrevion***:** IL: Interleukin; TNF-α: Tumor necrosis factor alpha. Data are presented as mean ± SD and median (p25 – p75). Adults correspond to the group from 45 to 59 years old and Older adults to the group from 60 to 74 years old. For comparison, Student's t was used for normal data and Mann–Whitney U for non-parametric data. p < 0.05 is considered significant

### Body composition and strength by excess fat groups

In the analysis of body composition and strength by groups with excess fat (WEF), not excess fat (NEF) and age group, no statistically significant differences were found for SMMI between adults and older adults NEF (9.1 ± 1.5 vs. 8.8 ± 1.3, *p* > 0.05). Likewise, the WEF adult group showed a significantly lower SMMI than the NEF adults (7.9 ± 0.8 vs. 9.1 ± 1.5, *p* < 0.001). On the other hand, WEF older adults showed significantly lower SMMI than NEF older adults (7.1 ± 7.1 vs. 8.8 ± 1.3, *p* < 0.001). Regarding strength, no statistically significant differences were found in the values between the adult and older adult NEF groups (28 ± 8 vs. 27 ± 8.6, *p* > 0.05). The WEF adult group showed significantly lower strength values than the NEF adults (22 ± 5 vs. 28 ± 8, *p* < 0.001). Likewise, WEF older adults had significantly lower strength than NEF older adults (17.9 ± 4.8 vs. 27 ± 8.6 *p* < 0.001). NEF older adults had higher SMMI values (8.8 ± 1.3 vs. 7.9 ± 0.8, *p* < 0.001) and strength (27 ± 8.6 vs. 22 ± 5, *p* < 0.05) when compared to WEF adults (Table 4).

**Table 4:** Body composition and strength according to the amount of body fat and age group

Variables	NEF (T1)		WEF (T3)	
	Adults *n* = 33	Older adults *n* = 45	Adults *n* = 33	Older adults *n* = 44
SMMI (kg/m^2^)	9.1 ± 1.5	8.8 ± 1.3	7.9 ± 0.8^a^	7.1 ± 7.1^b,c,d^
SMM (kg)	23 ± 5.1	22.8 ± 5.1	19 ± 2.8^e,g^	15.9 ± 1.8^f,h,i^
BMI (kg/m^2^)	28.7 ± 3.6	27.6 ± 3.2	34.4 ± 4.8^j,l^	32.6 ± 4.8^ k,m^
WC (cm)	94 ± 9	95 ± 13	105 ± 10^n,p^	101 ± 14^o,q^
Strength (kg)	28 ± 8	27 ± 8.6	22 ± 5^r,t^	17.9 ± 4.8^ s,u,v^

*NEF* Not excess fat, *WEF* With excess fat, *T1* Tertile 1, values less than or equal to the cut-off point of the 33 percentile of FM (%), *T3* Tertile 3, values greater than or equal to the 66 percentile cut-off point of FM (%), *FM* Fat mass, *SMMI* Skeletal muscle mass index, *SMM* Skeletal muscle mass, *BMI* Body mass index, *WC* Waist circumference. Data are presented as mean ± SD. Adults: 45 to 59 years old, Older adults: 60 to 74 years old. T1 NEF: cut-off point (adults ≤ 41 and older adults ≤ 45.2). T3 WEF: cut-off point (adults ≥ 46.1 and older adults ≥ 50.6)

One-way ANOVA with Tukey's post hoc test. **SMMI**: ^a^Adults (T1) *vs.* Adults (T3) *p* < 0.001; ^b^Adults (T1) *vs.* Older adults (T3) *p* < 0.001; ^c^Adults (T3) vs. Older adults (T3) *p* < 0.001; ^d^Older adults (T1) vs. Older adults (T3) *p* < 0.001. **SMM**: ^e^Adults (T1) *vs.* Adults (T3) *p* < 0.001; ^f^Adults (T1) *vs.* Older adults (T3) *p* < 0.001; ^g^Adults (T3) *vs.* Older adults (T1) *p* < 0.001; ^h^Adults (T3) vs. Older adults (T3), *p* < 0.05; ^i^Older adults (T1) *vs.* Older adults (T3) *p* < 0.001. **BMI:** ^j^Older adults (T1) *vs.* Adults (T3) *p* < 0.001; ^k^Older adults (T1) *vs.* Older adults (T3) *p* < 0.001; ^l^Adults (T3) *vs.* Older adults (T1) *p* < 0.001; ^m^Older adults (T1) *vs.* Older adults (T3) *p* < 0.001. **WC**: ^n^Adults (T1) vs. Adults (T3) *p* < 0.001; ^o^Adults (T1) *vs.* Older adults (T3) *p* < 0.05; ^p^Older adults (T1) *vs.* Adults (T3) *p* < 0.001; ^q^Older adults (T1) vs. Older adults (T3) *p* < 0.05. **Strength:** ^r^Adults (T1) *vs.* Adults (T3) *p* < 0.05; ^s^Adults (T1) *vs.* Older Adults (T3) *p* < 0.001; ^t^Older adults (T1) *vs.* Adults (T3) *p* < 0.05; ^u^Adults (T3) *vs.* Older adults (T3) *p* < 0.001; ^v^Older adults (T1) *vs.* Older adults (T3) *p* < 0.001

### Cytokines concentration by excess fat groups

No statistically significant differences were found between the WEF or NEF adult and older adult groups, except for IL-1, in which significantly higher levels were observed in NEF adults than NEF older adults (p < 0.01), likewise NEF adults. vs. WEF older adults (p < 0.01) (Table 5).

**Table 5:** Markers of inflammation according to the amount of body fat and age group

Variables	NEF (T1)		WEF (T3)	
	Adults *n* = 33	Older adults *n* = 45	Adults *n* = 33	Older adults *n* = 44
IL-1 ($\mu g/mL$)	14.7 (14.1 – 15.4)	9.4 (7.4 –11.2)^a^	11.7 (8.7 – 14.7)	8.0 (6.6 – 10)^b^
IL-6 ($\mu g/mL$)	6.1 (5.6 – 6.8)	7.1 (4.6 – 8.7)	7 (6.3 – 8.9)	7.5 (4.9 – 10.9)
IL-8 ($\mu g/mL$)	17.1 (15.1 – 22.2)	15.4 (11.6 – 52.8)	17.8 (14.1 – 26)	21.8 (13 – 72.6)
IL-10 ($\mu g/mL$)	4 (3.9 – 4.2)	3.8 (0.9 – 4.9)	3.8 (2.7 – 4)	3.4 (0.6 – 4.7)
TNF-α ($\mu g/mL$)	6.1 (5.9 – 6.3)	7.1 (2.7 – 8.8)	5.7 (4.5 – 6)	6.2 (1.1 – 7.8)

*NEF* no excess fat, *WEF* with excess fat, *T1* Tertile 1, values less than or equal to the cut-off point of the 33 percentile of FM (%), *T3* Tertile 3, values greater than or equal to the 66 percentile cut-off point of FM (%), *IL* interleukin, *TNF-α* Tumor necrosis factor alpha. Data are presented as mean ± SD. Adults: 45 to 59 years old, Older adults: 60 to 74 years old. T1 NEF: cut-off point (adults ≤ 41 and older adults ≤ 45.2). T3 WEF: cut-off point (adults ≥ 46.1 and older adults ≥ 50.6). One-way ANOVA with Tukey's post hoc test

**IL-1**: ^a^Adults (T1) *vs.* Older adults (T1) *p* = 0.002; ^b^Adults (T1) *vs.* Older adults (T3) *p* = 0.002

## Discussion

Human aging is accompanied by significant changes in body composition, characterized by an increase in the proportion of fat mass and a decrease in muscle mass and strength, and consequently an increased risk of sarcopenia and frailty [17]. However, these changes and risk related to aging can be modified through the adoption of healthy lifestyles, among which moderate physical exercise and healthy eating stand out. In contrast, obesity at early ages of the aging process (45 to 59 years) constitutes a significant risk factor for presenting sarcopenia [18–20]. For this reason, the purpose of the present study was to analyze the relationship between excess body fat with skeletal muscle mass index and strength in adults (45 to 59 years) compared to older adults (60 to 74 years).

Obesity is a public health problem in the world in the adult and older population. In Mexico, 74.2% of adults are overweight (39.1%) or obese (36.1), and 81.6% have abdominal adiposity. In this sense, the highest prevalence is observed in adults between 40 and 69 years of age [21, 22], which implies a higher risk of sarcopenia incidence, due to the additive effect of the association of obesity with the aging process [23].

Although it has been observed that serum levels of cholesterol and LDL increase until adulthood and decrease with aging, some factors, such as environmental (diet and physical exercise), hormonal, sleep disorders, psychological (distress), socioeconomic level and chronic consumption of some medications, can influence the lipid profile levels of older adults [24]. In this sense, it is more common to report a gradual age-related increase in plasma concentrations of triglycerides, cholesterol and LDL, while HDL remains unchanged. Likewise, it has been observed that HDL isolated (in vitro) from older people shows a lower cellular cholesterol clearance capacity compared to serum HDL levels from adults aged 20 to 30 years [25, 26]. In our study, a statistically significant increase in serum levels of LDL and HDL cholesterol was observed in the group of older adults, which contrasts with what was reported in the study carried out by Feng et al. (2020) in the Chinese population, who observed inconsistent changes by sex in relation to age [27]. This supports the point that environmental factors, including lifestyle (diet, physical exercise and sleep), are determinants of serum levels of the lipid profile regardless of age. In this regard, it is important to highlight that lipid metabolism is related to the pathophysiology of several chronic non-communicable diseases including sarcopenias [28].

On the other hand, higher body weight was observed in the adult population compared to that of older adults, which is consistent with what was reported in the National Health and Nutrition Survey of Mexico 2018 [29], however, FM (%) was significantly higher in older adults. In this regard, one of the main factors related to overweight and obesity in older adults is a sedentary lifestyle, the prevalence of which is significantly high, as reported in the systematic review carried out by Harvey et al. (213), who found a sedentary lifestyle prevalence of 67% in people over 60 years of age, characterized by physical inactivity of more than 8.5 h per day [30]. Likewise, in another systematic review and meta-analysis carried out by Silveira et al. (2022), found a prevalence of sedentary behavior of 31% (95% CI, 23 to 41%), and physical inactivity of 43% (95% CI, 31 to 55%) whith a significant associations between obesity and sedentary behavior (OR 1.45, 95% CI, 1.21 to 1.75) and physical inactivity (OR 1.52, 95% CI, 1.23 to 1.87) [31].

The relationship between obesity and the risk of sarcopenia is controversial, in this regard, it has been pointed out that excess body fat increases the risk of sarcopenia, due to greater oxidative stress (OxS), chronic inflammation (CI), insulin resistance, lipotoxicity and fatty infiltration in muscle cells (myosteatosis), which occurs in obesity, causing muscle atrophy [32, 33]. However, the concept of "obesity paradox" has been proposed in older adults, to point out the possible protective effect of obesity for the onset of sarcopenia. In this sense, in a systematic review and meta-analysis carried out by Liu et al. (2023), found that obesity was associated with 34% reduced risk of sarcopenia (OR = 0.66, 95% CI 0.48–0.91; p < 0.001) [34].

In our study, significantly higher SMMI (kg/m2) and strength were observed in the adult group compared to older adults, consistent with changes in body composition and risk of sarcopenia relative to aging [35]. However, as has been pointed out, excess FM (%) can cause loss of SMM and strength, regardless of aging, due to the negative effect that adipose tissue has on the body, such as increased OxS, CI, resistance to insulin and myosteatosis [32, 33]. In this regard, in the present study it was observed that the WEF adult and older adult groups had significantly lower strength and SMMI values than the NEF subgroups (adults and older adults). However, the NEF adult and older adult groups did not show significant differences in SMMI and strength values (p > 0.05), which suggests that excess FM (%) has a greater influence on SMM and strength than greater age. On the contrary, in a study conducted in adult men from Iran, a higher BMI was found to be an independent protective factor for the occurrence of sarcopenia [36], the result of which is consistent with the so-called “obesity paradox” [34], whose interpretation is controversial. In this sense, it has been pointed out that the muscle mass of obese people presents myosteatosis and therefore a certain degree of sarcopenic obesity, and consequently poor muscle function, regardless of the amount of muscle mass [32, 33]. On the other hand, our findings show that excess FM (%) in adults is equated to the effect of increasing age in older adults, so it can be assumed that excess FM (%) is a more relevant risk than aging per se for lose SMM and decrease strength. In this sense, we must consider that our study focused on the analysis of the relationship of excess body fat by age groups considering the cut-off points of the tertiles of body fat and we are not comparing obese and non-obese groups.

Regarding the relationship of inflammation markers with the loss of muscle mass, the importance of the inflammageing process has been highlighted as a pathophysiological mechanism of sarcopenia, linked to obesity and dysbiosis; the pharmacological indication of anti-inflammatory drugs has even been suggested for prevention and/or control of sarcopenia [37, 38]. Although in our study we only observed significantly higher levels of IL-1 in the NEF and WEF adult groups compared to older adults, the similarity in the concentration of the other markers (IL-6, IL-8, IL-10 and TNF-α) between the NEF and WEF adult and older adult groups, suggest that FM (%) largely determines the levels of said markers regardless of age, supporting the proposal that excess fat may be a factor similar to or of greater weight than older age for sarcopenia.

Finally, it is important to note among the main limitations of the study, the cross-sectional design, the sample size is not representative, in addition to the fact that markers of oxidative stress were not evaluated. For this reason, it is necessary to carry out more longitudinal and intervention studies, in which all hallmarks of aging linked to sarcopenia are considered [39], to have a comprehensive knowledge of the pathophysiology of this disease.

## Conclusion

Our findings suggest that excess fat mass has a significantly greater relationship than aging per se with skeletal muscle mass and strength. However, it is necessary to carry out longitudinal and intervention studies to confirm our results, considering a comprehensive analysis of all characteristics related to aging (hallmarks) linked to sarcopenia.

## Data Availability

The study data are available upon reasonable request to the corresponding author.
